# Socio-economic and demographic determinants affecting participation in the Swedish cervical screening program: A population-based case-control study

**DOI:** 10.1371/journal.pone.0190171

**Published:** 2018-01-10

**Authors:** Gudrun Broberg, Jiangrong Wang, Anna-Lena Östberg, Annsofie Adolfsson, Szilard Nemes, Pär Sparén, Björn Strander

**Affiliations:** 1 Department of Obstetrics and Gynaecology, Institute of Clinical Science, Sahlgrenska Academy, University of Gothenburg, Gothenburg, Sweden; 2 The Regional Cancer Centre, Western Health Care Region, Gothenburg, Sweden; 3 Närhälsan Primary Care, Western Health Care Region, Skövde, Sweden; 4 Department of Medical Epidemiology and Biostatistics, Karolinska Institute, Stockholm, Sweden; 5 Public Dental Service, Region Västra Götaland, Sweden; 6 Department of Behavioural and Community Dentistry, Institute of Odontology, Sahlgrenska Academy, University of Gothenburg, Gothenburg, Sweden; 7 The School of Health and Medical Sciences, Örebro University, Örebro, Sweden; 8 The Centre for Women’s, Family and Child Health, Faculty of Health Sciences, Buskerud & Vestfold University College, Kongsberg, Norway; 9 Swedish Hip Arthroplasty Register, Gothenburg, Sweden; Bharathidasan University, INDIA

## Abstract

**Background:**

Cervical screening programs are highly protective for cervical cancer, but only for women attending screening procedure.

**Objective:**

Identify socio-economic and demographic determinants for non-attendance in cervical screening.

**Methods:**

**Design**: Population-based case-control study.

**Setting:** Sweden.

**Population:** Source population was all women eligible for screening. Based on complete screening records, two groups of women aged 30–60 were compared. The case group, non-attending women, (N = 314,302) had no smear registered for 6–8 years. The control group (N = 266,706) attended within 90 days of invitation.

**Main outcome measures**: Risk of non-attendance by 9 groups of socioeconomic and demographic variables.

**Analysis**: Unadjusted odds ratios (OR) and OR after adjustment for all variables in logistic regression models were calculated.

**Results:**

Women with low disposable family income (adjOR 2.06; 95% confidence interval (CI) 2.01–2.11), with low education (adjOR 1.77; CI 1.73–1.81) and not cohabiting (adjOR 1.47; CI 1.45–1.50) were more likely to not attend cervical screening. Other important factors for non-attendance were being outside the labour force and receiving welfare benefits. Swedish counties are responsible for running screening programs; adjusted OR for non-participation in counties ranged from OR 4.21 (CI 4.06–4.35) to OR 0.54 (CI 0.52–0.57), compared to the reference county. Being born outside Sweden was a risk factor for non-attendance in the unadjusted analysis but this disappeared in certain large groups after adjustment for socioeconomic factors.

**Conclusion:**

County of residence and socio-economic factors were strongly associated with lower attendance in cervical screening, while being born in another country was of less importance. This indicates considerable potential for improvement of cervical screening attendance in several areas if best practice of routines is adopted.

## Introduction

Cervical cancer is considered to be a preventable disease [1]. The incidence of and mortality from cervical cancer have decreased in countries with organised screening programs and the disease has now become relatively rare in Sweden [2]. Participation is one prerequisite for screening program success, and non-attendance has been shown to be the foremost risk factor for cervical cancer related to the screening program [3]. Low socio-economic status is associated with increased incidence of and mortality from cervical cancer [4]. There is some evidence that low education, older age and living alone are related to advanced cervical cancer stages at diagnosis, due to non-attendance in cervical screening [5]. Furthermore, immigrants generally have lower rates of attendance [6]. Previous studies have also found that attendance in screening is lower among older women [7, 8]; single women [9, 10]; women with low socio-economic status [11], including a low education level [8, 12]; and women with low use of health care [8, 13]. In order to create conditions for more equitable care, it is therefore important to examine factors related to non-attendance in cervical screening. The aim of this study was to identify socio-economic and demographic determinants for attendance in cervical screening in Sweden.

## Methods

We conducted a case-control study based on extensive population data linked to the Swedish National Cervical Screening Registry [14].

### Cervical screening program in Sweden

The organised cervical screening program was initiated in the late 1960s in Sweden. The national guidelines, issued by the Swedish National Board of Health and Welfare and valid at the time of this study, recommended Papanicolaou (Pap) smears at three-year intervals for women aged 23–50 and at five-year intervals for women aged 51–60 [15]. Women aged over 60 were not invited to the cervical screening program in Sweden because regular screening until age 60, with no abnormal smears, was considered to entail a low risk of cervical cancer. The regional screening programs are administered independently in the 21 Swedish counties and practical routines vary, although the basic national guidelines concerning age limits and screening intervals are generally adhered to. All Swedish citizens and permanent resident have a personal identification number (PIN), and women eligible for screening are identified by this number. Having a PIN thus is a requisite for receiving an invitation. The invitation usually includes the time and place for an appointment. In Sweden screening smears are taken at Antenatal Health Clinics by midwives. In some counties information in several languages are attached to the invitation. There are also differences across counties when it comes to issuing reminders if a woman does not show up after invitation, as well as in availability and opening hours, invitation design, offer of scheduled appointments and the possibility to reschedule an appointment over the Internet. Most counties, but not all, charge a fee for screening; the amounts vary.

### Data sources

The source population was identified through the Swedish Total Population Register [16], which also contains information about place of residence, country of birth and date of immigration. The unique personal identity number (PIN) assigned to every resident in Sweden was used for record linkage between the registers [17]. Information on invitations to attend cervical screening and attendance was retrieved from the Swedish National Cervical Screening Registry (NKCx) [14]. This register has complete coverage since 1993 and, among other information, contains data about all Pap smears taken in Sweden, both within and outside the organised screening program. The register also includes data on all screening invitations issued by the Swedish counties to their residents. From the National Patient Register [18], we retrieved information on total hysterectomies. Information on cohabiting status, disposable family income, employment status, unemployment benefits, social benefits and education level was retrieved from the Longitudinal Database on Health Insurance and Labour Market Studies (LISA) [19], held by Statistics Sweden. The database contains annual registers since 1990 and includes all individuals 16 years of age and older that were registered in Sweden as of December 31 each year.

### Study sample

**Fig 1:**
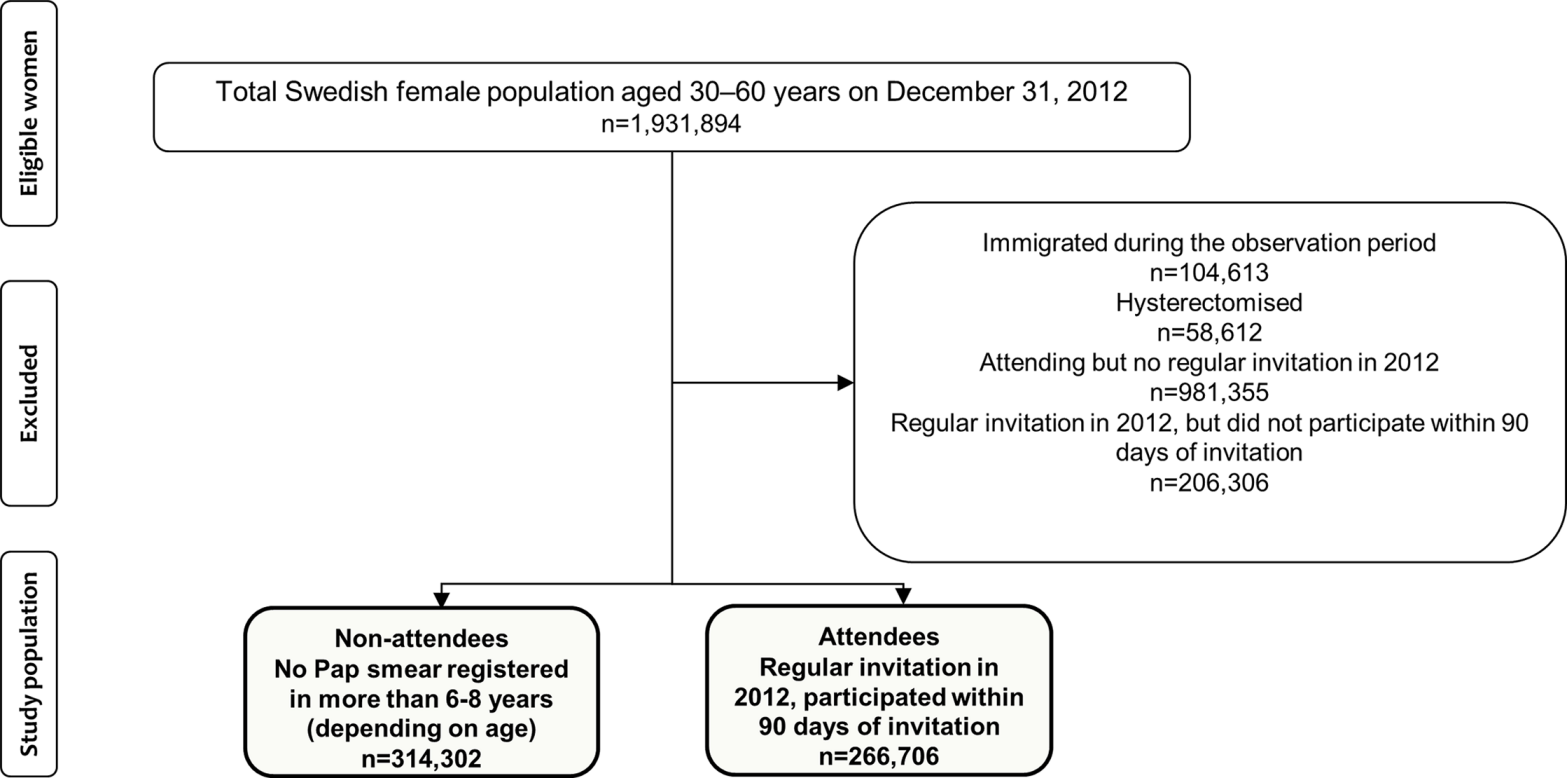
Flowchart of enrolment in the study.

We compared women who had not participated in the screening program for a long time with women who participated after receiving a regular invitation. The source population consisted of the entire Swedish female population between 30 and 60 years of age on December 31, 2012. (Fig 1). To become a case, non-attendance from regular screening for at least 6 years was a requisite, which is the reason for not including women below 30. The upper age limit corresponds with the upper limit for screening [15].

#### Selection of cases

Women without a registered Pap smear in the last six years if aged 30–53 years, in the last seven years if aged 54 years and in the last eight years if aged 55–60 years (until December 31, 2012) were designated “non-attendees”, corresponding to age-dependent screening intervals.

#### Selection of controls

Women who had received a regular invitation (i.e. not a reminder) between January 1 and December 31, 2012, and who were screened within 90 days after being invited, were designated “attendees”.

#### Exclusions

Women who had immigrated to Sweden during the study period, and thereby could not fulfil criteria for cases, or those who had undergone total hysterectomy were excluded.

### Determinants

Non-attendees and attendees were compared regarding the following determinants: Age was stratified into six categories (30–34, 35–39, 40–44, 45–49, 50–54 and 55–60). Additional demographic factors considered in the analysis were country of birth, divided into regions based on the United Nations’ Population Division [20], and the county of residence in Sweden. Six categorical variables were used as socio-economic indicators. Disposable family income for the income year 2010 was divided into high (>50.111 €), medium (24.222–50.111 €) and low (<24.222 €) (exchange rate: € 1 = 9 SEK). In labour force was defined as women who had declared income to the tax authorities in 2012, classified as yes or no. Unemployment benefits was defined as full-time or part-time unemployment compensation in 2010, classified as yes or no. Due to low numbers, we used the combined category welfare benefits classified as yes or no. Yes was equal to receiving either social welfare or housing benefits or both in 2010. Education level (the highest formal education attained in 2012) was classified into three categories according to the Swedish education system: primary school = low (≤ 9 years), secondary school = medium (10–12 years) and higher = high (>12 years). Cohabitation with a partner in 2010 was categorised as yes or no.

The immigrant women included in the study were from 163 different countries, grouped in 19 of the 21 United Nations’ Population Division regions. Melanesia was merged with Australia and New Zeeland, due to low numbers.

### Statistical analysis

The associations between socio-economic and demographic variables and the outcome measure, i.e. non-attendance, were calculated in logistic regression models with odds ratios (OR), and their corresponding 95% confidence intervals (95% CI), as outcome measure. As all categories of variables we were interested in exhibited significant association with non-attendance in the univariate analysis, we decided to keep them in the multivariate model.

Statistical analyses were run on complete samples; observations with one or more missing values were removed. Due to the large sample size, no imputations were performed. Statistical tests were run in R 3.1.0.

### Ethics

Ethical approval was obtained by the Regional Ethics Committee in Stockholm (Dnr 98–002, 02–556 and 2011/921–32). The database information was decoded after the necessary linkages were concluded. The PIN was used to link each participant’s data in the different registers, after which a unique identification number was assigned to each woman by Statistics Sweden, replacing the PIN. No names or PINs were thus provided to the researchers by Statistics Sweden, ensuring confidentiality.

The study conforms to the Strobe Statement and the checklist for Observational Studies in Epidemiology [21]. There is no published Core Outcome Set (COS) applicable to this study. There was no patient involvement in obtaining funding or designing the study.

## Results

The source population of Swedish women, aged 30–60 years, consisted of 1,931,894 individuals on December 31, 2012. Of these, non-attendees constituted 314,302 (16%) women who had not participated in screening during the 6-8-year follow-up period. The attendees constituted 266,706 (14%) women who had received a regular invitation during 2012 and who did attend within 90 days. During this year, a total of 473,012 women received a regular invitation and 206,306 of these did not attend within 90 days and were excluded from the study. A total of 104,613 women had immigrated during the 6–8-year follow-up period and 58,612 women had a history of total hysterectomy and were likewise excluded. More than half of the source population, 981,355 women, had participated in screening at least once during the follow-up period, but were not due for screening in 2012 and had not received a regular invitation that year (Fig 1). The characteristics of non-attendees and attendees are shown in Table 1.

**Table 1:** Characteristics of non-attendees (N = 314,302) and attendees (266,706) and missing data.

		Non-attendees			Attendees	
		n	Proportion of all (%)	Proportion with available data (%)	n	Proportion of all (%)
*Age groups (years)*						
	30–34	60,631	19.3	19.3	36,581	13.7
	35–39	56,964	18.1	18.1	45,200	16.9
	40–44	53,469	17.0	17.0	49,997	18.7
	45–49	52,829	16.8	16.8	52,875	19.8
	50–54	44,575	14.2	14.2	37,502	14.1
	55–60	45,834	14.6	14.6	44,551	16.7
	Data missing	0	0.0		0	1.0
*Place of birth*						
	Sweden	182,903	58.2	58.2	219,008	82.1
	Northern Europe except Sweden	25,030	8.0	8.0	7,758	2.9
	Central and Eastern Europe	19,015	6.0	6.1	5,881	2.2
	Southern Europe	12,133	3.9	3.9	6,552	2.5
	Western Europe	7,975	2.5	2.5	1,713	0.6
	North America	3,825	1.2	1.2	516	0.2
	Central America	753	0.2	0.2	343	0.1
	Caribbean	429	0.1	0.1	159	0.1
	South America	5,550	1.8	1.8	2,683	1.0
	North Africa	2,180	0.7	0.7	709	0.3
	East Africa	9,680	3.1	3.1	2,172	0.8
	Southern Africa	353	0.1	0.1	52	0.0
	West Africa	1,185	0.4	0.4	438	0.2
	West Asia	15,709	5.0	5.0	7,940	3.0
	South Central Asia	11,431	3.6	3.6	4,293	1.6
	Southeast Asia	8,487	2.7	2.7	4,640	1.7
	East Asia	6,516	2.1	2.1	1,711	0.6
	Australia/New Zeeland/Melanesia	999	0.3	0.3	107	0.0
	Data missing	149	0.0		31	0.0
*County*						
	Stockholm	72,449	23.1	23.3	56,422	21.2
	Uppsala	42,486	13.5	13.6	7,044	2.6
	Södermanland	10,365	3.3	3.3	8,362	3.1
	Östergötland	12,784	4.1	4.1	9,746	3.7
	Jönköping	7,448	2.4	2.4	11,714	4.4
	Kronoberg	7,405	2.4	2.4	3,292	1.2
	Kalmar	4,436	1.4	1.4	9,610	3.6
	Gotland	2,985	0.9	1.0	1,379	0.5
	Blekinge	2,905	0.9	0.9	4,073	1.5
	Skåne	39,233	12.5	12.6	25,908	9.7
	Halland	13,034	4.1	4.2	8,411	3.2
	Västra Götaland	42,567	13.5	13.7	53,159	19.9
	Värmland	4,015	1.3	1.3	4,501	1.7
	Örebro	6,910	2.2	2.2	10,402	3.9
	Västmanland	7,908	2.5	2.5	6,524	2.4
	Dalarna	6,361	2.0	2.0	9,241	3.5
	Gävleborg	6,236	2.0	2.0	9,837	3.7
	Västernorrland	5,035	1.6	1.6	8,924	3.3
	Jämtland	3,522	1.1	1.1	3,902	1.5
	Västerbotten	7,223	2.3	2.3	7,880	3.0
	Norrbotten	6,099	1.9	2.0	6,361	2.4
	Data missing	2,896	0.9		14	0.0
*Disposable family income*, *quartile group*						
	High >50,111 €	63,775	20.3	21.1	125,565	47.1
	Medium 24,222–50,111 €	92,727	29.5	30.7	96,693	36.3
	Low <24,222 €	145,059	46.2	48.1	44,346	16.6
	Data missing	12,643	4.0		102	0.0
*In labour force*						
	Yes	138,095	43.9	59.7	226,796	85.0
	No	93,081	29.6	40.3	38,664	14.5
	Data missing	83,126	26.4		1,246	0.5
*Unemployment benefits*						
	No	196,366	62.5	84.9	233,669	87.6
	Yes	34,810	11.1	15.1	31,791	11.9
	Data missing	83,126	26.4		1,246	0.5
*Welfare benefits*						
	No	240,106	76.4	89.5	254,681	95.5
	Yes	28,040	8.9	10.5	11,602	4.4
	Data missing	46,156	14.7		423	0.2
*Education*						
	High >12 years	108,219	34.4	39.3	122,081	45.8
	Medium 10–12 years	118,683	37.8	43.1	119,076	44.6
	Low ≤9 years	48,482	15.4	17.6	24,352	9.1
	Data missing	38,918	12.4		1,197	0.4
*Cohabiting*						
	Yes	106,313	33.8	46.0	179,346	67.2
	No	124,861	39.7	54.0	86,113	32.3
* *	Data missing	83,128	26.4		1,247	0.5

Missing data was more frequent in non-attendees, and consisted mainly of socioeconomic variables (Table 1). Most women with missing data were born outside Sweden.

The mean age of the non-attendees and the attendees was 43.7 years (SD = 8.76) and 44.8 years (SD = 8.41), respectively. The results of the univariate and the multivariate analysis for both groups are shown in Table 2.

**Table 2:** Crude odds ratios and adjusted odds ratios with 95% confidence intervals for non-participation in screening program, according to categories of variables. The analyses were run on complete samples.

		Non-attendees/ Attendees	OR (95% CI)	Adjusted* OR (95% CI)
		n		
*Age groups (years)*				
* *	30–34	38,549/35,966	*ref*	
* *	35–39	34,661/44,642	0.72 (0.71–0.74)	0.91 (0.89–0.93)
* *	40–44	35,411/49,584	0.67 (0.65–0.68)	0.90 (0.88–0.92)
* *	45–49	38,874/52,539	0.69 (0.68–0.70)	0.91 (0.89–0.93)
* *	50–54	34,173/37,276	0.86 (0.84–0.,87)	1.08 (1.06–1.11)
* *	55–60	35,545/44,346	0.75 (0.73–0.76)	0.92 (0.90–0.94)
*Place of birth*				
* *	Sweden	151,538/218,036	*ref*	
* *	Northern Europe except Sweden	10,423/8,158	1.84 (1.78–1.89)	1.36 (1.32–1.41)
* *	Central and Eastern Europe	8,893/5,189	2.47 (2.38–2.55)	1.64 (1.57–1.70)
* *	Western Europe	2,723/1,646	2.38 (2.24–2.53)	1.96 (1.83–2.09)
* *	Southern Europe	7,688/6,415	1.72 (1.67–1.78)	1.24 (1.19–1.29)
* *	North America	923/494	2.69 (2.41–3.00)	1.93 (1.71–2.17)
* *	Central America	331/331	1.44 (1.24–1.68)	0.94 (0.79–1.11)
* *	Caribbean	193/153	1.82 (1.47–2.24)	1.02 (0.81–1.28)
* *	South America	3,038/2,631	1.66 (1.58–1.75)	1.02 (0.97–1.08)
* *	North Africa	1,240/673	2.65 (2.41–2.91)	1.37 (1.24–1.52)
* *	West Africa	574/411	2.01 (1.77–2.28)	0.95 (0.83–1.09)
* *	Central Africa	220/148	2.14 (1.74–2.64)	1.08 (0.86–1.36)
* *	East Africa	4,974/1,868	3.81 (3.63–4.04)	1.73 (1.64–1.84)
* *	Southern Africa	101/51	2.85 (2.04–3.99)	1.99 (1.38–2.86)
* *	West Asia	10,631/7,650	2.00 (1.94–2.06)	1.01 (0.97–1.04)
* *	South-Central Asia	6,221/4,193	2.14 (2.05–2.22)	1.30 (1.24–1.36)
* *	Southeast Asia	4,751/4,524	1.51 (1.45–1.58)	0.87 (0.83–0.91)
* *	East Asia	2,523/1,677	2.17 (2.03–2.30)	1.38 (1.29–1.48)
* *	Australia/New Zeeland/Melanesia	228/105	3.12 (2.48–3.94)	2.13 (1.66–2.74)
*County*				
	Västra Götaland	28,484/52,718	*ref*	
	Stockholm	64,103/55,908	2.12 (2.08–2.16)	2.33 (2.28–2.38)
	Uppsala	13,158/6,789	3.59 (3.47–3.71)	4.21 (4.06–4.35)
	Södermanland	7,282/8,295	1.63 (1.57–1.68)	1.66 (1.60–1.72)
	Östergötland	9,716/9,671	1.86 (1.80–1.92)	1.96 (1.90–2.03)
	Jönköping	5,121/11,624	0.82 (0.79–0.85)	0.88 (0.84–0.91)
	Kronoberg	5,283/3,253	3.01 (2.87–3,15)	3.49 (3.33–3.67)
	Kalmar	3,391/9,565	0.66 (0.63–0.68)	0.69 (0.66–0.72)
	Gotland	1,893/1,368	2.56 (2.39–2.75)	2.88 (2.67–3.11)
	Blekinge	2,416/4,053	1.10 (1.05–1.16)	1.15 (1.08–1.21)
	Skåne	33,687/25,668	2.43 (2.38–2.48)	2.47 (2.41–2.53)
	Halland	5,057/8,291	1.13 (1.09–1.17)	1.24 (1.19–1.29)
	Värmland	3,548/4,472	1.47 (1.40–1.54)	1.36 (1.29–1.43)
	Örebro	5,565/10,321	1.00 (0.96–1.03)	1.02 (0.99–1.06)
	Västmanland	5,979/6467	1.71 (1.65–1.78)	1.74 (1.67–1.82)
	Dalarna	2,800/9,170	0.57 (0.54–0.59)	0.54 (0.52–0.57)
	Gävleborg	4,714/9,789	0.89 (0.86–0.93)	0.89 (0.86–0.93)
	Västernorrland	3,514/8,880	0.73 (0.70–0.76)	0.75 (0.72–0.78)
	Jämtland	2,263/3,875	1.08 (1.02–1.14)	1.14 (1.07–1.20)
	Västerbotten	5,245/7,851	1.24 (1.19–1.28)	1.43 (1.37–1.48)
	Norrbotten	3,994/6,325	1.17 (1.12–1.22)	1.22 (1.16–1.27)
*Disposable family income*, *quartile groups*				
* *	High > € 50,111	55,174/125,107	*ref*	
* *	Medium € 24,222–50,111	76,848/95,975	1.82 (1.79–1.84)	1.30 (1.28–1.33)
* *	Low < € 24,222	85,191/43,271	4.46 (4.40–4.53)	2.06 (2.01–2.11)
*In labour force*				
* *	Yes	136,425/226,463	*ref*	
* *	No	80,788/37,890	3.54 (3.49–3.59)	2.15 (2.11–2.19)
*Unemployment benefits*				
* *	No	182,655/232,636	*ref*	* *
* *	Yes	34,558/31,717	1.39 (1.37–1.41)	0.85 (0.83–0.86)
*Welfare benefits*				
* *	No	192,820/252,994	*ref*	
* *	Yes	24,393/11,359	2.82 (2.75–2.88)	1.52 (1.48–1.56)
*Education*				
* *	High >12 years	79,149/121,379	*ref*	
* *	Medium10-12 years	97,578/118,710	1.26 (1.25–1.28)	1.27 (1.25–1.28)
	Low ≤ 9 years	40,486/24,264	2.56 (2.51–2.61)	1.77 (1.73–1.81)
*Cohabiting*				
* *	Yes	100,913/178,706	*ref*	
* *	No	116,300/85,647	2.41 (2.38–2.43)	1.47 (1.45–1.50)

*Adjusted for all other variables

Women in the youngest age group (30–34 years) were more likely to be non-attendees than those in all other age groups in the univariate analysis. This effect persisted after adjustment for all other covariates in all age groups, except age 50–54 years. Women in this latter age group had a slightly higher chance of not participating (adjOR 1.08; 95% CI 1.06–1.11) than those in the youngest age group, after adjustments.

To be born in any country other than Sweden was associated with a higher risk of non-attendance in the univariate analysis. After adjustment, the risk remained increased for Europe, North America, Northern Europe (adjOR 1.36; 95% CI 1.32–1.41), Central and Eastern Europe (adjOR 1.64; 95% CI 1.57–1.70), Western Europe (adjOR 1.96; 95% CI 1.83–2.09), Southern Europe (adjOR 1.24; 95% CI 1.19–1.29) and North America (adjOR 1.93; 95% CI 1.71–2.17). However, women in some immigrant groups participated to an extent equalling that of Swedish women in the adjusted analysis: Central America (adjOR 0.94; CI 0.79–1.11), South America (adjOR1.02; 0.97–1.08), West Africa (adj0.95; 0.83–1.09), Central Africa (adjOR1.08; CI 0.86–1.36) and West Asia (adjOR1.01; CI 0.97–1.04). Women from South-East Asia were less likely not to participate than Swedish-born women (adjOR 0.87; 95% CI 0.83–0.91), after adjustment for covariates.

Regionally, there was a wide variation in participation between Swedish counties. Compared with the reference county (Västra Götaland), ORs for not attending ranged from 4.21 (95% CI 4.06–4.35) in Uppsala county to 0.54 (95% CI 0.52–0.57) in Dalarna county, after adjustments.

The mean annual disposable family income was € 34,523 (SD = 47,924) in non-attendees and € 55,204 (SD = 65,794) in attendees, a relative difference of 60%. Women with the lowest disposable income (< €24 222) were twice as likely not to participate, compared to those in the highest income category (> €50 111) (adjOR 2.06; 95% CI 2.01–2.11), after adjustment for all other variables. Women with medium income were 30% more likely not to participate (adjOR 1.30; 95% CI 1.28–1.33) (Table 2). Women outside the labour force were twice as likely not to participate in screening than women with jobs (adjOR 2.15; 95% CI 2.11–2.19). Women receiving unemployment benefits were less likely to be non-attendees than women who were not benefits recipients (adjOR 0.85; 95% CI 0.83–0.86), after adjustment for other covariates. On the other hand, women receiving welfare benefits were found to be about 50% more likely not to participate, after adjustments (adjOR 1.52; 95% CI 1.48–1.56). Women with up to 9 years of education were almost 80% more likely not to participate, compared to women with more than 12 years of education (adjOR 1.77; 95% CI 1.73–1.81), while those with 10–12 years of education were almost 30% more likely not to participate (adjOR 1.27; 95% CI 1.25–1.28) (Table 2).

Cohabitation, including marriage, affected non-attendance in the multivariate model, in which single women had a higher chance of not attending than cohabiting women (adjOR 1.47; 95% CI 1.45–1.50).

## Discussion

In this large population-based case-control study, we found that county of residence, income, being in the labour force, country of birth, education, living with a partner, and receiving welfare benefits were all independently related to non-attendance in cervical screening. To obtain a clear distinction in attendance, we compared two well-defined groups at each end of the spectrum: those who had not participated for six to eight years and women who attended screening soon after being invited.

The Swedish National Board of Health and Welfare issues guidelines on cervical screening but each county runs its screening program autonomously. The county of residence remained one of the strongest determinants for participation after adjustment for all other covariates. These results concur with the coverage by county reported in the Swedish National Cervical Screening Registry [22]. It is encouraging that a well-run screening program can, to a high extent, compensate for the negative impact on participation of low socioeconomic status and immigration, and this finding should have implications for screening programs outside Sweden as well. Thus, there is obviously potential for improvement in many Swedish counties. Our results show that women with low socio-economic status and women born in other countries are at a disadvantage in relation to the Swedish screening program. Several strategies, such as invitation with a scheduled appointment [23], reminder letter [24, 25] and telephone reminder [25, 26], have been proven to increase participation. Offering women a Pap smear when they visit a gynaecologist for other reasons [13, 27] and Human papillomavirus (HPV) self-tests have also been proven to increase participation [28–32]. Collaboration with local doulas in immigrant areas can identify barriers to attend and facilitate interventions to increase participation in cervical screening[33]. Making these adaptations to existing screening programs in each county might increase participation and provide more equitable access to screening. Västra Götaland, one of the three most populated counties in Sweden, was chosen as a reference as it consists of both urban and rural areas. In this county and in others with high participation, some of the above-mentioned measures to increase participation have already been implemented, as well as a re-booking system on the Internet.

Being born outside Sweden had significant impact on the probability of non-attendance [6, 8]. A somewhat surprising finding was, however, that women from Southeast Asia, South America, and West Asia (including the Middle East), large groups living in Sweden, participated to the same, or greater, extent than Swedish-born women, after adjustment for socio-economic and other demographic factors. This suggests that participation in screening, at least for women born in these areas, is not so much hampered by language or cultural barriers as by socioeconomic factors.

While some studies have found that high age is a predictor of non-participation [7, 8], we found that young women (aged 30–34) had a slightly increased risk of non-participation, compared to women at older ages. This is in accordance with published quality register data [28]. The same data show, however, that screening coverage among the youngest age group in Sweden, not included in this study, has increased in recent years. One reason for this might be the attention that cervical cancer has received in the media in association with HPV vaccination.

We assessed several indicators of socio-economic status in order to obtain a better estimate of women’s social situation in relation to cervical screening attendance. In accordance with other studies, socio-economic status was a strong predictor of non-attendance [8, 29, 30]. Income turned out to be the strongest factor determining participation. This somewhat surprising result was unaltered after adjustment for the other variables, possibly explained by the fee charged in most counties. However, the fee is quite limited (€ 10–20) and the only Swedish county that does not charge a fee, Stockholm, is among the areas with the lowest participation. Only one randomised study has examined the importance of a fee for attendance. This recent study was conducted in a socioeconomically deprived area in Sweden and failed to reveal any difference in attendance between women who paid a modest fee and those receiving an offer of a Pap smear free of charge [31].

This study has several strengths. It is very large, with cases and controls derived from the entire Swedish female population aged 30–60 years. It relies on clinical and socio-demographic, instead of self-reported, data, compiled from high-quality national registers containing reliable individual information on cervical screening attendance, socioeconomic status and immigration status.

This study also has limitations. It should be noted that ORs cannot be interpreted as relative risks, due to the high prevalence of the outcome. However, the relative importance of the exposures and statistical significance calculations are not affected. There were missing data for some variables, which might have led to biased results and false interpretations of associations. E.g.,data on socio-economic factors were missing mainly in non-attending immigrant women; it can be assumed that this is more likely to yield an underestimation of the difference between groups. Our analysis were made on complete samples without imputation, and differences in completeness imply that interpretation of the diverse level of association across variables should be done with some caution. E.g. the fact that information on disposable family income was more complete than other socio-economic factors might explain why it had the strongest association with non-participation.

This study is limited to the factors available in the registers and does not explore all possible factors that could influence non-attandance. Differences across counties have to be analysed further. Organisational issues as providing invitations in several languages, opening hours, reminders, providing fixed appointments with easy re-scheduling, offering HPV selfsampling for non-attendees should be considered. Distance to service provider has shown to be of importance in breast cancer screening uptake in UK [34] Switzerland [35] and Denmark [36] The impact of distance in cervical screening in Sweden with a well distributed network of antenatal care units is not known and needs to be studied.

The cervical screening program in Sweden is obviously fails to offer service on equal terms to the population, as differences in attendance are highly dependent on socio-economic status and county, responsible for screening organisation. There is still a major need for more research on how groups with lower attendance should be targeted.

## Conclusion

We found that county of residence and socio-economic factors (Lower family income, lower education, being outside labour force, receiving welfare benefits, not cohabiting) were strongly associated with lower attendance in cervical screening, while being born in another country was of less importance. This indicates considerable potential for improvement of cervical screening attendance in several areas if best practice of routines is adopted.
